# A Systematic Review of Metal Composite Bone Grafts in Preclinical Spinal Fusion Models

**DOI:** 10.3390/biomimetics10090594

**Published:** 2025-09-05

**Authors:** Christian Rajkovic, Mahnoor Shafi, Naboneeta Sarkar, Vaughn Hernandez, Liwen Yang, Timothy F. Witham

**Affiliations:** Department of Neurosurgery, Johns Hopkins School of Medicine, Baltimore, MD 21210, USA; crajkov1@jhu.edu (C.R.);

**Keywords:** spinal fusion, metals, bone graft, arthrodesis, strontium, magnesium

## Abstract

Successful arthrodesis is a crucial factor in spinal fusion surgery, maximizing the likelihood of improved quality of life. The incorporation of metals into bone grafts has been demonstrated to enhance fusion rates through various osteoinductive and osteoconductive pathways. A systematic review was conducted to investigate the utility of metal composite bone grafts in promoting arthrodesis in spinal fusion preclinical studies. PubMed/MEDLINE was queried to identify studies investigating metal composite bone grafts in animal models of spinal fusion. Non-spinal fusion animal models were excluded. Risk of bias was assessed using the SYRCLE risk of bias tool. After screening a total of 1554 articles, 17 articles were included in our review. Metal composite bone grafts with bioactive agents had significantly greater fusion rates than metal composite only bone grafts (*p* < 0.001) and similar fusion rates compared to non-metal comparator bone grafts (*p* = 0.172). Bone grafts containing strontium and magnesium had the greatest fusion rates compared to other metals and had significantly greater fusion rates than those of silicon-containing bone grafts (*p* = 0.02 and *p* = 0.04, respectively). Bone quality and bone volume percentages of fusion masses formed by metal composite bone grafts were enhanced via the addition of bioactive agents such as stem cells, rhBMP-2, autograft, and poly (lactic-co-glycolic acid). The adverse event rate was 3.0% in all animal surgeries. Metal composite bone grafts show promise as osteoinductive agents to promote arthrodesis in spinal fusion, and their osteoinductive capability is enhanced with the synergistic addition of osteogenic factors such as stem cells and autograft.

## 1. Introduction

Spinal fusion is becoming an increasingly common operation to treat debilitating degenerative, infectious, neoplastic, and traumatic diseases of the spine [1]. The clinical judgement to pursue a spinal fusion operation is often multifactorial, with an emphasis on regaining or preserving patient quality of life and neurologic function. Successful arthrodesis, or the formation of continuous bone across adjacent vertebral segments, portends a positive prognosis for the patient, including postoperative pain relief, neurological recovery, and reduced morbidity. Re-establishment of physiologic spinal anatomy and biomechanics often decompresses focal areas of neurologic compromise and eliminates maladaptive modeling of the spine that serves as the impetus for chronic inflammation and pain [2]. However, rates of pseudoarthrosis, or failed fusion, remain relatively high, between 5 and 35%, and up to 60% of patients report persistent chronic back pain after spinal fusion [1,3]. 

Several methodologies have been investigated to improve spinal fusion rates, including novel bone grafts composed of small molecules, biomaterials, and biologics such as recombinant human bone morphogenetic protein-2 (rhBMP-2). Historically, autologous iliac crest bone graft has been chosen for patients undergoing spinal fusion due to its cellular osteogenic and native osteoinductive and osteoconductive properties [4]. However, the harvesting process for these grafts comes with considerable risks, including donor site pain, blood loss, infection, injury, and fracture [5]. Therefore, investigation into synthetic bone graft materials is ongoing to limit these risks while preserving successful arthrodesis rates with new materials. The current gold standard for synthetic bone grafts to promote arthrodesis is Medtronic’s Infuse^®^ product, an absorbable collagen sponge infused with rhBMP-2 [6]. However, rhBMP-2 is associated with its own risks, such as heterotopic ossification, edema, radiculitis, and a theoretical increase in malignancy, which is currently unsubstantiated in the literature [5,7,8]. Modern synthetic ceramics have also shown promise as novel osteoinductive agents. MagnetOs Easypack Putty utilizes a tricalcium-phosphate (TCP) and hydroxyapatite (HA) composite with unique submicron surface topography to induce spinal fusion and was shown to have a successful arthrodesis rate of 94.4% in a sample of 36 patients receiving transforaminal lumbar interbody fusion [9]. However, the advent of MagnetOs and other novel ceramics is in the early stages of clinical development, and therefore, further investigation of novel bone graft technologies is required.

Metals have shown promise as osteoinductive and osteoconductive agents that stimulate bone formation with several biomimetic properties that mimic native human bone. Several trace metals are naturally incorporated into physiologic bone formation, such as sodium, magnesium, zinc, silicon, and strontium, and the similar electronegative properties of these metals may serve as an acceptable substitute in the osteogenic process for calcium [10]. Magnesium is an essential cofactor used for the synthesis of ATP, DNA, and RNA in osteoblastic precursors [11]. Strontium promotes natural bone formation through both the stimulus of osteoprotegerin and the inhibition of receptor activator of nuclear factor kappa ligand (RANKL)-mediated osteogenesis [12]. Further, magnesium and copper have been shown to increase angiogenesis in animal models of novel bone graft substitutes [13,14]. Surface topography properties of heavy metals also contribute to their biomimetic properties, as porous titanium and tantalum have been demonstrated as osteoconductive agents in several orthopedic settings through deposition of native osteoinductive factors on their surfaces with a similar elastic modulus to native bone [15,16]. 

In spinal fusion procedures, metals, particularly titanium, are often used as interbody implants for their osteoinductive and osteoconductive properties. Porous tantalum has been investigated as a bone graft substitute due to its similar mechanical properties to cancellous bone. A systematic review by Hanc et al. (2015) observed low rates of implant failure and heterogeneous rates of fusion in clinical studies with this material [17]. Further, titanium mesh cages have often been used with or without autograft or allograft materials to promote spinal fusion. A randomized control trial by Thome et al. (2006) observed similar fusion rates and clinical outcomes in patients who received either iliac crest autograft or rectangular titanium meshes in anterior discectomy and fusion procedures [18]. Modern technologies have further improved these materials with a 3D-printed porous tantalum cage investigated by Liang et al. (2025), demonstrating effective clinical outcomes in a pilot study of posterior lumbar interbody fusion cases [19]. While these studies demonstrate the effectiveness of metals as implants, the investigation of novel, metal-composite bone grafts for spinal fusion requires further study.

This systematic review seeks to investigate metals as potential synergistic biomaterials in composite bone grafts to induce osteogenesis in preclinical animal models of spinal fusion. We hypothesize that incorporation of metals into bone graft substitutes will promote greater fusion rates and bone quality both with and without the presence of auxiliary agents such as rhBMP-2 or autologous bone graft. We will also investigate adverse effects and toxicities associated with relevant metals incorporated in the investigated bone grafts.

## 2. Literature Review

### 2.1. Study Design and Search Strategy

**Figure 1:**
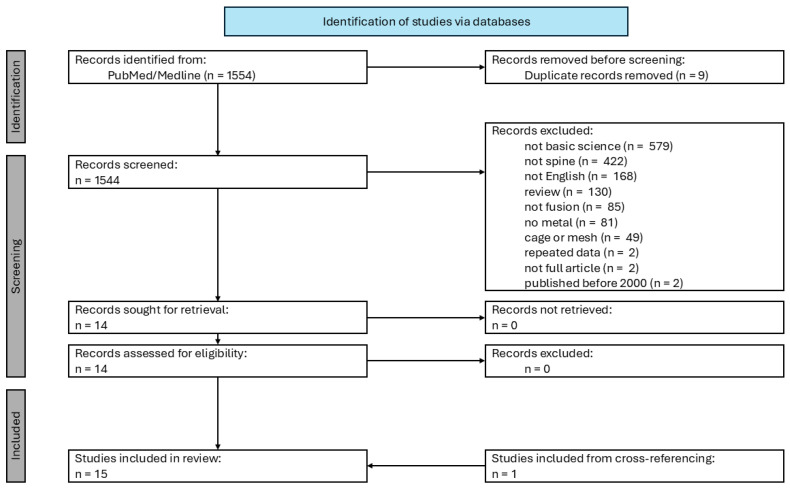
The preferred reporting items for systematic reviews and meta-analyses (PRISMA) flowchart of search results.

A systematic review of PubMed/MEDLINE was performed in accordance with the Preferred Reporting Items for Systematic Reviews and Meta-Analyses (PRISMA) (Figure 1) [20]. The PubMed/MEDLINE database was searched for articles relevant for metal composite bone grafts in animal models of spinal fusion according to the following PICOS criteria: population: laboratory animals, intervention: metal composite bone grafts in a spinal fusion animal model, comparator: non-metal-containing bone grafts or cage materials, outcomes: fusion rate, fusion bone quality, and adverse events, and study type: in vivo animal studies. No protocol was prospectively registered for this study. Full search terms are outlined in Appendix A. This review was registered with PROSPERO (ID: 1136094).

### 2.2. Eligibility Criteria

Screened articles were included based on the following inclusion criteria: (1) basic science studies, (2) studies with an animal model of spinal fusion, and (3) studies investigating metal additives to bone graft material to promote spinal fusion. Studies were excluded based on the following exclusion criteria: (1) non-English studies, (2) abstracts and unpublished studies, (3) clinical studies, (4) reviews, (5) articles investigating non-spinal bone fusion, (6) articles investigating metal cages or meshes in the absence of bone grafts, (7) articles investigating bone grafts composed of solely ceramic and/or organic biomaterials, (8) animal models investigating adjunct medical pathologies in addition to spinal fusion, and (9) studies published before the year 2000.

### 2.3. Selection Process and Screening

Search query and subsequent deduplication were performed by C.R. Articles were then independently screened by authors (C.R. and M.S.) for basic science studies investigating metal composite bone grafts in animal models of spinal fusion based on our aforementioned inclusion and exclusion criteria. Bibliographies of included articles were also screened for relevant articles. Screening was confirmed by one additional reviewer (N.S.).

### 2.4. Data Extraction and Data Outcomes

Study design characteristics such as the animal model, bone graft materials, spine segment operated on, surgery type, and length of study were recorded for all included articles. Primary outcomes evaluated included fusion rate of experimental animals, bone volume percentage (BV%) of masses, and histologic analysis of fusion masses. In studies where multiple follow-up points were described, data points were taken from the latest postoperative follow-up. Successful fusion was defined by data extractors as continuous bridging of bone from caudal to cephalad ends of adjacent transverse processes (TPs). Secondary outcomes investigated included surgical adverse events and mortality, implant toxicity, and implant corrosion. Data that was only reported graphically was extracted using webplotdigitizer version 5.0 (https://automeris.io/WebPlotDigitizer/; accessed on 23 June 2025) software to estimate mean and standard deviation values.

### 2.5. Risk of Bias Assessment

Risk of bias assessment was carried out independently by two reviewers (M.S. and V.H.) using the Systematic Review Center for Laboratory Animal Experimentation (SYRCLE) Risk of Bias Tool [21]. This assessment tool utilizes ten questions to assess study selection bias, performance bias, detection bias, attrition bias, reporting bias, and other biases. An answer of “yes” to a particular question confers a low risk of bias for that bias type, an answer of “unclear” to a particular question confers an unclear risk of bias for that bias type, and an answer of “no” to a particular question confers a high risk of bias for that bias type.

### 2.6. Statistical Analysis

All statistical analysis was performed using GraphPad Prism 10 software (GraphPad, La Jolla, CA, USA). Fisher’s exact testing was performed for comparison of fusion rates and adverse events across bone graft materials. Bone volume percentages of fusion masses were not compared across studies, and all comparisons reported for this outcome are extracted from comparisons already performed within each independent included study. Further, to assess the sensitivity and heterogeneity of our results, subgroup analysis regarding metal-only and metal bone grafts with additives was performed with respect to controls in the included studies. These subgroups were also further stratified to compare specific metals with adjunctive bioactive agents and specific metals without adjunctive bioactive agents. Studies lacking one or more investigated outcomes were excluded from all statistics involving that outcome. A *p*-value less than 0.05 was considered statistically significant. All tables were synthesized using Microsoft Excel^®^.

## 3. Results

### 3.1. Baseline Characteristics of Included Studies

After screening a total of 1554 articles, 15 articles were included in our review, comprising 507 total experimental animals. One study was published in two parts as two separate articles [22,23]. Animal models of included studies were as follows: rabbit (n = 5), sheep (n = 4), rat (n = 3), dog (n = 1), and goat (n = 1). Posterolateral lumbar fusion was performed in 11 studies, with one of those studies also performing discectomies. The remaining three studies investigated anterior lumbar interbody fusion (n = 1) and anterior cervical discectomy and fusion (ACDF) (n = 2) for their animal models. Metals investigated in the included studies were magnesium (n = 5), titanium (n = 3), strontium (n = 3), silicon (n = 2), and tantalum (n = 1). Comparison fusion materials included autograft (n = 7), autograft with poly (lactic-co-glycolic acid) (PLGA) (n = 1), a titanium cage (n = 1), a silicon hydride ceramic cage (n = 1), HA ceramic (n = 1), HA ceramic with bisphosphonate (n = 1), and tricalcium phosphate ceramic (n = 1). Adjunct bioactive agents investigated with metal composite bone grafts included autograft (n = 4), demineralized bone matrix (DBM) (n = 2), bone marrow stem cells (BMSCs) (n = 2), rhBMP-2 (n = 1), NaOH and HCl treatment (n = 1), adipose stem cells (ADSCs) (n = 1), platelet-rich plasma (PRP) (n = 1), poloxamer hydrogel (n = 1), PLGA (n = 1), and polydeoxyribonucleotide (PDRN) (n = 1). Average latest follow-up for included studies was 15.1 ± 12.3 weeks (range: 6–52 weeks). However, a follow-up of 52 weeks was only present in one study comparing a porous titanium-nickel composite bone graft to a titanium–aluminum–vanadium (Ti-Al-V) cage with autologous bone graft [23]. Eliminating this outlier, the average latest follow-up was 12.3 ± 7.1 weeks (range: 6–26 weeks). Study designs of the included articles are outlined in Table 1.

SYRCLE risk of bias assessment was performed on all included studies and is outlined in Table 2. Randomization of experimental animals was only reported in five studies, and no study had blinded group allocation of operated animals due to the distinctive nature of the bone grafts and surgical procedures investigated in the included articles. Similarly, knowledge of the intervention was also not blinded in any of the investigated studies. Detection bias was also relatively high, as outcome assessment blinding was only performed in 6/14 unique studies. However, attrition and reporting bias were deemed to be low in 14/14 and 11/14 studies, respectively.

### 3.2. Arthrodesis Rates

Overall fusion rate for all animals among included studies was 82% or 268 of 326 animals that had fusion rate reported as an outcome. Fusion rate among animals treated with metal composite only bone grafts was 62%. Metal composite bone grafts with adjunct bioactive agents had a significantly greater fusion rate compared to metal composite grafts alone at 88% (*p* < 0.001). Comparator bone grafts had a fusion rate of 83% (*p* = 0.004 compared to metal-only composite grafts and *p* = 0.172 compared to metal composite bone grafts with adjunct bioactive agents).

Comparing specific metals in the investigated composite bone grafts, strontium bone grafts had the highest overall fusion rate at 100% (18/18 animals), compared to 89% (95/107 animals) with magnesium composite grafts, 76% (26/34 animals) with titanium composite grafts, 72% (21/29 animals) with silicon composite grafts, and 12.5% (⅛ animals) with tantalum composite grafts. Comparisons of fusion rates between metals showed significant differences between magnesium-containing grafts and silicon-containing grafts (*p* = 0.04), strontium-containing grafts and silicon-containing grafts (*p* = 0.02), and strontium-containing bone grafts and titanium-containing bone grafts (*p* = 0.04). Additionally, tantalum had significantly decreased fusion rates compared to all other metals (*p* < 0.001 for magnesium and strontium; *p* = 0.002 for titanium and *p* = 0.003 for silicon). The fusion rate of animals receiving only autograft was 84% (70/83 animals), which was only significantly greater than that of tantalum (*p* < 0.001). In a study by Assad et al. (2003) comparing a porous titanium-nickel composite bone graft to a comparator Ti-Al-V cage with autologous bone graft, all four animals in each group investigated at 52 weeks demonstrated complete spinal fusion and were included in the group analyses [23]. However, it should be noted that earlier time points of 12 weeks follow-up observed 3/6 animals in the metal composite group fused compared to 0/6 animals in the cage group, and at a slightly later follow-up of 24 weeks, all 6/6 animals fused in the metal composite group fused compared to 0/6 in the cage group.

Among metal composite bone grafts that did not receive any adjunctive bioactive agents, strontium bone grafts had the highest fusion rate at 100% (18/18) animals compared to 85.7% (12/14) animals in titanium bone grafts, 37.5% (3/8 animals) in silicon bone grafts, 12.5% (1/8 animals) in magnesium bone grafts, and 0% (0/4 animals) in tantalum bone grafts Statistical comparison between metals in this group revealed significantly higher fusion rates with strontium compared to magnesium (*p* < 0.001), silicon, and tantalum (*p* < 0.001 for all comparisons) and significantly higher fusion rates with titanium compared to magnesium (*p* = 0.002) and tantalum (*p* = 0.005) with borderline significance compared to silicon (*p* = 0.05). All strontium composite bone grafts investigated in the included studies did not have adjunctive bioagents, and therefore, no strontium bone grafts were included in the analysis of metal composite bone grafts with adjunctive agents.

Among metal composite bone grafts with adjunctive bioactive agents, magnesium bone grafts had the highest fusion rate at 94.9% (94/99 animals), followed by silicon bone grafts at 85.7% (18/21 animals), titanium bone grafts at 70% (14/20 animals), and tantalum bone grafts at 25% (1/4 animals). Magnesium-containing bone grafts with adjunctive bioactive agents also had significantly higher fusion rates than both titanium bone grafts with adjunctive bioactive agents (*p* = 0.003) and tantalum bone grafts with adjunctive bioactive agents (*p* = 0.001), while silicon-containing bone grafts with adjunctive bioactive agents only had significantly greater fusion rates than tantalum bone grafts with adjunctive bioactive agents (*p* = 0.03). Adjunctive agents used in magnesium-containing bone grafts were iliac crest bone graft (n = 91 animals), demineralized bone matrix (n = 34 animals, PRP (n = 8 animals), PLGA (n = 75 animals), and PDRN (n = 25 animals). Adjunctive agents used in titanium-containing bone grafts were iliac crest bone graft (n = 15 animals) and NaOH and HCl solution (n = 5 animals). Adjunctive agents used in silicon-containing bone grafts were a poloxamer hydrogel (n = 5 animals), iliac crest bone graft (n = 16 animals), and BMSCs (n = 8 animals). The only adjunctive agent used in tantalum-containing bone grafts was rhBMP-2 (n = 4 animals).

### 3.3. Bone Quality of Formed Bone Masses

**Figure 2:**
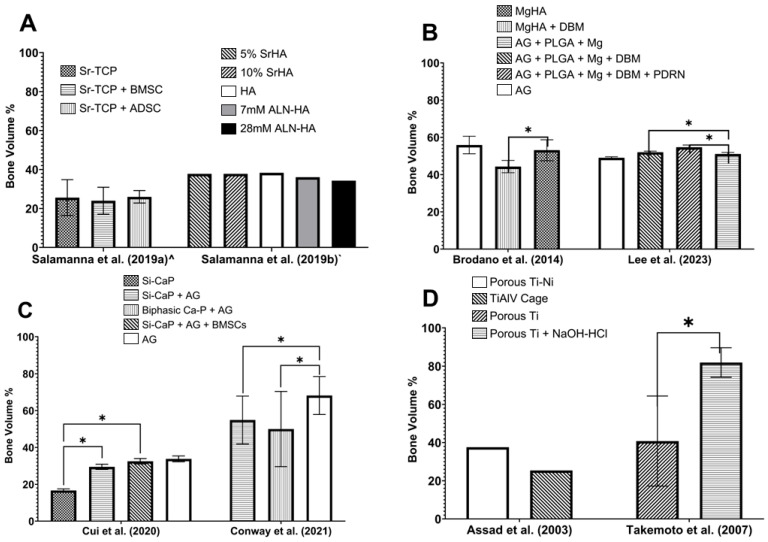
Bone Volume Percentages (BV%s) of Fusion Masses. Mean and standard deviation BV%s are plotted for eight included studies. (**A**) BV%s for studies investigating strontium-composite bone grafts [29,30]. (**B**) BV%s for studies investigating magnesium-composite bone grafts [28,35]. (**C**) BV%s for studies investigating silicon-composite bone grafts [33,34]. (**D**) BV%s for studies investigating titanium-composite bone grafts [22,26]. ‘*’ denotes *p* < 0.05 for intra-study comparisons.

BV%s were reported as outcomes in 9/15 of the included articles, and BV%s for titanium, magnesium, strontium, and silicon bone grafts with their comparator grafts are described in Figure 2. Treatment of a tantalum composite bone graft with rhBMP-2 demonstrated a nonsignificant increase in proportional bone volume growth compared to a control tantalum composite bone graft in a goat model of ACDF with 8 animals described by Sidhu et al. (2001) [24]. Assad et al. (2023) observed a steady increase in bone quality from 21.4% to 37.6% over a 12-month follow-up period following PLF with a porous titanium-nickel composite graft compared to a significantly lower and more static bone quality change from 22.7% to 25.4% (*p* < 0.05) over the same follow-up period following PLF with a Ti-Al-V cage [22]. A porous titanium implant in a canine model of ALIF investigated by Takemoto et al. (2007) achieved a similar BV% of 40.8 ± 23.6% at a twelve-week follow-up that significantly increased to 81.9 ± 7.7% (*p* < 0.05) with preoperative treatment of the graft using NaOH and HCl [26].

The effect of magnesium on the bone quality of fusion masses, denoted by BV%, was varied among the included studies. Investigation of a MgHA composite bone graft by Brodano et al. (2014) in a sheep model of PLF at 24 weeks follow-up demonstrated a BV% of 53.1 ± 5.6% that was lowered to 44.3 ± 3.3% when DBM was added to the graft, significantly lower than the 55.9 ± 4.7 BV% achieved by autograft in the same model (*p* < 0.05) [28]. However, the addition of magnesium-DBM to an autograft substitute treated with PLGA and PDRN achieved a significantly higher BV% of 54.7 ± 1.2% compared to the BV% of 49 ± 0.6% achieved by autograft alone in a rat model of PLF by Lee et al. (2023), suggesting a synergistic effect of magnesium on bone formation when paired with these novel biologics [35].

BV% was reported as an outcome in two studies investigating strontium-containing bone grafts. A rat model of PLF by Salamanna et al. (2019) observed an average BV% of 37.8 (IQR: 36.4–39.0) and 37.8 (IQR: 35.4–40.2) for 5% SrHA and 10% SrHA, respectively, achieving similar BV%s to HA alone (38.3%) and HA treated with 7 mM (36.1%) and 28 mM (34.3%) alendronate [30]. A subsequent study by the same author observed a BV% of 25.6% eight weeks following PLF with a Sr-TCP bone graft in a rat model, which was not statistically different from the 24.0% and 26.0% achieved when the Sr-TCP graft was treated with BMSCs and ADSCs, respectively [29].

Lastly, silicon-containing composite bone grafts and their effect on BV% were described in two rabbit models of PLF, one by Cui et al. (2021) and one by Conway et al. (2021) [33,34]. Cui et al. (2021) [33] investigated a Si-TCP bone graft at 12 weeks postoperatively and achieved BV% of 16.7 ± 0.8% which was significantly increased to 29.5 ± 1.4% with the addition of autologous bone graft (*p* < 0.05) and further significantly increased 32.5 ± 1.9% with the addition of autologous bone graft and BMSCs to the Si-TCP graft (*p* < 0.05). However, the 32.5 ± 1.9 BV% achieved with the Si-TCP and autologous bone graft treated with BMSCs was not significantly different than the 33.8 ± 1.6 BV% acquired 12 weeks after fusion with autograft alone. Similarly, Conway et al. (2021) investigated a hydrogel-Si-TCP biomaterial as a supplement to autologous bone graft and found that both this bone graft and biphasic calcium phosphates with autograft acquired significantly lower BV%s than autograft alone (54.9 ± 13.0% vs. 68.2 ± 10.3%, *p* < 0.05 and 50.0 ± 20.4% vs. 68.2 ± 10.3%, *p* < 0.05, respectively) [34].

Histologic analysis of fusion masses was performed in all of the included articles. Metal composite bone grafts were noted to stimulate deposition of osteoid on decorticated TPs in 12/15 included studies. Forms of bone identified noted on histology of fusion masses in these studies were mature cancellous and cortical bone in nine studies [23,26,28,29,30,32,33,34], cortical bone only in one study [31], and woven bone in one study [25]. Significant fibrous tissue was also noted in the fusion masses of metal composite bone grafts investigated in five studies [24,25,26,29,33]. Persistent metallic debris was noted on histology in two studies [24,25], and peri-implant inflammation was noted in three studies [25,34,35]. Apoptotic or necrotic tissue at the fusion site was noted in one study [25].

### 3.4. Adverse Events and Toxicity

A total of 15 adverse events were reported in 507 animal surgeries performed in our included studies. The adverse events occurred in the goat model by Sidhu et al. (2021) [24], the rabbit model by Cunningham et al. (2002) [25], the sheep model by Assad et al. (2003) [23], the rabbit model by Van Eps et al. (2021) [32], and the rabbit model by Cui et al. (2021) [33]. The most common adverse events were unknown intraoperative mortality (n = 9) and mortality during iliac crest harvest (n = 4). No metal-related adverse events occurred. Metal toxicity was investigated by four of the included studies, and none observed any toxic systemic or local effects from the metal composite grafts [23,27,36]. Investigation of a porous titanium-nickel composite in a sheep model by Assad et al. (2003) showed no significant change in nickel content in all major organ systems compared to control animals [23]. Similarly, no changes in serum studies or blood metal ion content were observed following use of a magnesium autograft described by Kaya et al. (2007) and a strontium–hardystonite–gahnite graft described by Newsom et al. (2023) [27,28,36].

## 4. Discussion

To the authors’ knowledge, this is the first systematic review investigating metal composite bone grafts in animal models of spinal fusion. We observed that metal composite bone grafts with auxiliary bioactive agents fused at similar rates compared to comparator bone grafts and significantly greater rates compared to bone grafts composed of metal composites alone. Bone grafts composed of strontium or magnesium were the most promising in promoting arthrodesis compared to the other investigated metals. Further, subgroup analysis also revealed that strontium-containing bone grafts achieved the highest arthrodesis rates among metal bone grafts that did not have adjunctive agents, and magnesium-containing bone grafts achieved the highest arthrodesis rates among metal bone grafts that did have adjunctive agents. Further, the addition of the investigated bioactive agents to most of the metal composite bone grafts described in our included studies was correlated with a significantly greater proportional growth of bone tissue compared to bone tissue formed by comparator or metal composite only bone grafts. Therefore, preclinical literature of spinal fusion molecules suggests promising evidence for metal composites as osteoinductive supplements to novel bone graft designs. 

### 4.1. Strontium as Novel Osteoinductive Biomaterial

The biological basis for the induction of bone formation with strontium is primarily through a dual mechanism of stimulating osteoblasts and inhibiting osteoclast-mediated bone resorption through the RANKL, osteoprotegrin, and calcium-sensing receptor (CaSR) pathways [37,38]. As a naturally occurring physiological ion, strontium primarily exists in native bone either in the cortical bone lattice or hydroxyapatite phase as a substitute for calcium [39]. During the bone healing process, strontium binds to the CaSR on osteoblastic precursors to upregulate osteoprotegrin expression and osteoblast proliferation through NF-kB, MAPK, and Wnt intracellular signaling while downregulating RANKL expression [40,41]. Subsequent osteoprotegrin expression by activated osteoblasts competitively inhibits RANKL binding on osteoclasts to further inhibit RANKL signaling and consequent bone resorption [42]. However, the stimulatory effect of strontium on osteogenesis is dependent on both the effective dose of strontium itself and the local concentration of calcium [37]. When in physiologic excess of approximately 10 mmol, strontium has been observed to have toxic inhibitory effects on osteoblastic proliferation, and when accompanied by low local doses of calcium, strontium has been shown to have an inhibitory effect on bone regeneration [43,44,45].

In vivo preclinical models of bone healing and formation have also used strontium in a variety of biomaterials to stimulate osteogenesis. Strontium-infused bioactive glasses have shown promise in promoting bone formation in various bone defect models. In these materials, strontium likely has a synergistic effect with the other osteoinductive metals present in the glass to reduce proinflammatory signaling while enhancing the material’s biomimetic compatibility [46]. In two independent models of femoral condyle defect, release of strontium from bioactive glass materials was shown to promote superior bone regeneration compared to non-strontium-infused bioactive glasses [47,48]. In TCP and HA ceramics, strontium has shown effective biomimetic capacity in substituting for calcium in these agents to enhance bone mineralization in vivo [49]. When augmented with agents such as stem cells and novel bioactive polymers such as polycaprolactone (PCL), a sustained release profile and synergistic osteoinductive effect of strontium further enhances bone mineralization and osteogenic differentiation. These novel bioactive polymers, such as PCL and PLGA, have also been used independently of ceramics with strontium in hydrogel, microsphere, and microparticle delivery modalities to promote cellular and osseous integration of these materials with improved osteoblastic differentiation and matrix mineralization [37,50,51]. Therefore, the fusion rate of 100% in the relatively small sample size of 18 animals in this study demonstrates an expansion of evidence of strontium as a novel osteoconductive material from simple bone healing to complex spinal fusion procedures.

Strontium has also been used in several clinical applications to improve bone health. Strontium ranelate has been in clinical use since the early 2000 s for the treatment of osteoporosis and is thought to improve bone quality in osteoporosis. A ten-year longitudinal study described by Reginster et al. (2012) observed a sustained antifracture effect with preserved bone mineral density in osteoporotic patients taking strontium ranelate [52]. Strontium has also been investigated in the use of spinal fractures to improve bone healing. A pilot study of 23 patients by Cheung et al. (2005) observed clinical improvement in patients who received strontium-containing cement during vertebroplasty for osteoporotic fractures [53]. Further, a study of 38 female patients with severely (>40%) compressed A2- and A3/AO-type thoracolumbar fractures by Korovessis et al. (2018) observed similar pain outcomes, segmental kyphosis, and vertebral body height ratios in these patients who received short percutaneous pedicle screw fixation plus polyetheretherketone implant with SrHA compared to the implant with standard polymethacrylate [54].

### 4.2. The Role of Magnesium in Enhancing Osteogenesis in Bone Tissue Engineering

Magnesium has also been implicated in several biologic pathways as a regulator of osteogenesis. In undifferentiated BMSCs, magnesium has been shown to enhance vascular endothelial growth factor (VEGF) expression through intracellular HIF-2α signaling to improve vascularization of new bone, and in osteoblastic BMSC precursors, VEGF expression is enhanced with simultaneous magnesium-induced expression of peroxisome proliferator-activated receptor gamma coactivator (PGC)-1α, a coactivator of VEGF transcription [55]. Magnesium has been observed to stimulate β-catenin nuclear translocation and subsequent Wnt signaling to induce extracellular mineralization and alkaline phosphatase expression [56]. Similarly, magnesium-induced activation of β-catenin has also been associated with enhanced activation of MAPK/ERK signaling and subsequent proliferation of osteoblasts [57]. Mechanically, magnesium has a similar elastic modulus to native trabecular bone, imparting a biomimetic mechanical advantage to the material. However, supraphysiologic concentrations of magnesium have been associated with significant osteotoxicity, limiting osteoblastic differentiation, enhancing bone resorption, and inhibiting hydroxyapatite formation [58]. Further, the metallurgical properties of magnesium can leave magnesium-containing materials susceptible to excessive corrosion and formation of reactive oxygen species [59].

Previous literature provides extensive in vivo and in vitro evidence supporting the osteogenic capabilities of magnesium as an adjunctive metal added to novel bone graft materials. A study investigating the role of nanomaterials in osteoblast maturation confirmed the promise of utilizing magnesium nanoparticles to promote bone regeneration, evidenced by their ability to enhance osteogenic differentiation through inflammatory response modulation to improve alkaline phosphatase activity, bone volume, and mineral density [60]. Consistent with this finding, magnesium alloy-reinforced bioglass cement scaffolds in a rabbit radius defect model by Duan et al. (2020) demonstrated significantly improved bone filling, bone mineral density, and osteoblast density compared to bioactive glass alone, similar to the findings observed with strontium and bioactive glass [61]. An in vitro study by Kim et al. (2021) has also observed osteogenesis stimulated by magnesium hydroxide-incorporated PLGA scaffolds similar to what is observed by Lee et al. (2023) in this review [35,62]. Cells grown on this scaffold had 3–6x higher expression of essential osteogenic markers when magnesium hydroxide was added compared to scaffolds with PLGA alone. Further enhancement of osteogenic marker expression was also observed via the addition of rhBMP-2 and PDRN in a nanocomplex with the magnesium hydroxide and PLGA scaffolds, suggesting synergetic effects on osteogenic outcomes [62]. Transcriptome sequencing of murine osteoblast-like MC3T3-E1 cells treated with magnesium by Liu et al. (2020) demonstrated an upregulation of osteoblast differentiation and expression of platelet-derived growth factor to stimulate angiogenesis of human umbilical vein endothelial cells [63]. Magnesium has also been shown to exhibit antimicrobial properties through stimulation of reactive oxygen species formation and subsequent oxidative stress and rupture of bacterial membranes [64]. Therefore, the mechanisms by which magnesium promotes bone formation likely involve several osteogenic, angiogenic, anti-inflammatory, and antimicrobial pathways, suggesting the role of novel bioactive agents to supplement magnesium to promote bone formation in spinal fusion models described in this review.

### 4.3. Synergistic and Biomimetic Mechanisms of Metal-Induced Osteogenesis 

The bioactive agents discussed in this review have also been observed to synergistically act with metals to promote bone formation in non-spinal fusion preclinical studies. A potential weakness of metal-composite bone grafts is the absence of live, cellular osteogenic material or natural biomolecular osteoinductive components. Therefore, the addition of bioactive materials such as rhBMP-2, biopolymers, or stem cells may improve bone formation and quality achieved with novel metal-composite bone grafts as seen in this study.

rhBMP-2 is currently the gold standard for clinically approved osteoinductive growth factors promoting spinal fusion and has exhibited significant efficacy in forming bone in several preclinical and large clinical studies [65]. Because of the widespread use of titanium implants for orthopedic applications, rhBMP-2 coating of titanium implants has been investigated in several animal models with improved osseointegration of the implants and enhanced volume and quality of bone formation compared to titanium implants alone [66,67,68]. Magnesium has also been observed to improve rhBMP-2 binding to the BMP-2 receptors and enhance activation of the Smad pathway to improve bone regeneration [69]. Strontium has been observed to similarly activate the same BMP-2/Smad1 pathway implicated in rhBMP-2 stimulation of osteogenesis [70]. In vivo, a murine bone defect model by Quade et al. (2020) observed enhanced bone formation and quality with the addition of strontium to a rhBMP-2-infused mineral collagen scaffold [71]. Further, a rat calvarial defect model by Kim et al. (2021) examining rhBMP-2 use with a magnesium hydroxide composite bone graft with PLGA and PDRN observed an approximately 3.7-fold higher bone mineral density and significantly greater BV% compared to PLGA grafts alone [62].

PLGA has also been shown in several in vivo and in vitro studies to improve the effective bioavailability of metal composite biomaterials through an increased resistance to corrosion, improved biocompatibility, and a controlled release profile of the encapsulated metal [72]. Delivery of magnesium with PLGA microspheres in a rat calvarial defect model described by Yuan et al. (2019) resulted in sustained release of magnesium ions and a significant promotion in bone regeneration, suggesting a prolonged and enhanced osteoinductive effect of magnesium with PLGA-mediated release [73]. Similarly, PLGA microspheres have also been shown to stimulate osteogenic differentiation and proliferation of osteoblastic precursors when loaded with strontium ranelate or strontium ranelate-HA nanoparticles [74]. A composite scaffold with PLGA-incorporated Sr/Zn-HA nanoparticles has also been described by Hassan et al. (2020) as a novel, biomimetic biomaterial for bone tissue engineering [75]. A PLGA-Cage-like structure loaded with both strontium and magnesium-doped HA was observed to have a continuous release profile of Sr and Mg to facilitate osteoblastic proliferation through osteogenic and angiogenic pathways in vitro [76].

The investigation into novel delivery methods of stem cells or donor iliac crest bone graft with metals may further enhance bone formation with metal bone grafts, due to their acellular content. Several metals, such as strontium, magnesium, copper, titanium, silver, and chromium, have shown good biocompatibility with mesenchymal stem cells while helping facilitate osteogenic differentiation of these cells, similar to the histologic findings observed in this review [77,78,79,80,81,82,83]. Biological grafts such as autograft, allograft, and DBM simultaneously stimulate osteogenic, osteoinductive, and osteoconductive pathways of bone formation through their cellular material, physical scaffolding, and extracellular matrix proteins. Porous metals such as titanium or magnesium have been shown to provide a physical meshwork with a surface density that can facilitate bony ingrowth of cellular bone graft materials and native osteoblastic stem cells [84]. A microporous magnesium-calcium phosphate scaffold investigated by Wei et al. (2010) was shown to stimulate superior attachment and proliferation of fibroblast cells compared to both calcium phosphate controls and macroporous magnesium-calcium phosphate composites, highlighting the inherent osteoinductive nature of magnesium as a material as well as the osteoconductive potential of its surface topography [85]. When viewed collectively, the data and conclusions drawn from the literature support our study’s findings of the importance of bioactive agents in supplementing metal composite bone grafts to improve bone formation.

Several other reviews have also examined the osteoinductive capabilities of metals outside of spinal fusion. A review by Li et al. (2021) [86] investigating metal implants used in orthopedics examined the relative advantages and disadvantages of magnesium, strontium, and zinc as adjunct biomaterials in bone formation. In addition to the significant evidence of promoting osteogenesis and angiogenesis for all three of these materials, magnesium’s biomechanical properties and elastic modulus were observed to be most consistently similar to those of bone. Zinc was most often described as a bioactive coating for non-load-bearing applications to improve biocompatibility, and strontium has a consistent osteoinductive effect on bone remodeling through the enhanced differentiation of osteoblasts and inhibition of osteoclasts. Another review by Wang et al. (2017) [87] evaluated metals as a novel osteoinductive biomaterial in the context of modern bone graft substitutes for orthopedic and dental osteogenesis. Magnesium, strontium, silicon, zinc, copper, lithium, and cobalt have all shown preclinical promise as osteoinductive factors that stimulate bone formation; however, the cost, availability, and toxicity of the novel biomaterials used to optimally deliver these metals currently severely hamper their advancement to clinical use [87]. Despite these two review articles discussing the roles of bioactive metals in bone regeneration, their observations mainly focus on narrative descriptions of general bone formation and defect repair, lacking a systematic, quantitative synthesis of outcomes or specificity concerning spinal fusion. In comparison, our systematic review focuses on metal composite bone grafts in spinal fusion models while conducting statistical comparisons of arthrodesis and a succinct synthesis of fusion bone quality and adverse events observed with novel metal composite bone grafts. Further, the synergistic effects when combining clinically relevant bioactive agents with the metal composite grafts are also described, highlighting future directions for research to optimize synthetic bone grafts for spinal fusion.

### 4.4. Limitations

Although this systematic review was able to comprehensively evaluate metal composite bone grafts in preclinical models of spinal fusion, there are several inherent limitations present due to the nature of the animal models investigated and the translatability of the metal composite bone grafts and their materials. The study designs of the included articles introduced some inherent selection and performance bias due to the knowledge investigators had of the bone graft and surgical procedure each animal was receiving. However, this limitation is likely inherent to the different physical appearances of metal composite graft materials and the subject matter of the animal models investigated in this study.

The designs of the spinal fusion models investigated in this study were also heterogeneous concerning the animal studied and the spinal level operated on, in addition to the various bone grafts described. Therefore, evaluation of the bone grafts was limited to the radiographic fusion rate on microCT and histologic bone quality rather than any motor, neurologic, or biomechanical benefits of the fusion due to the limited translatability of these parameters across animal studies or to humans. Further, to maximize the external validity of each study in our analysis, data from comparator groups were also extracted to demonstrate intra-model effectiveness of each graft relative to comparator grafts. Comparisons of bone quality between different metals were also not performed in any included study from the literature; therefore, it is difficult to extrapolate the relative osteoinductivity of the investigated metals to each other, rather than presenting the absolute effectiveness of each graft demonstrated by individual studies.

Finally, variation in follow-up existed in our studies even after excluding a 52-week outlier. While this variation in follow-up could be a potential confounder for arthrodesis rates given that a longer follow-up could select for higher fusion rates, a follow-up timeline of 6 to 26 weeks is consistent with clinical follow-up in human patients. Further, the timeline for spinal fusion in animals is variable depending on the graft and animal, with most small animal models requiring 6–8 weeks for fusion, while larger animal models have reported up to twenty weeks for successful fusion with significant variability between studies [88,89,90]. However, given the significant heterogeneity of experimental design and animal models of our included studies, analysis of the arthrodesis rates described at the latest follow-up was chosen to remain consistent with the expected timeline of arthrodesis of each investigated animal model. Therefore, the arthrodesis rates presented in this study should be interpreted as a representation of the effectiveness of these grafts in promoting successful spinal fusion in each model, not an assessment of their absolute osteoinductive capability.

Future studies may seek to evaluate the osteoinductivity of different metals in a single model of spinal fusion or evaluate various bioactive agents in a single metal composite in a single model of spinal fusion. Further, these studies could be supplemented with in vitro data that propose a specific mechanism for the investigated graft beyond prior established mechanisms of osteoinductivity of the graft’s components.

## 5. Conclusions

Metals show promise as osteoinductive and osteoconductive agents to stimulate spinal fusion as independent graft materials and additives to novel osteoinductive biomaterials. In particular, metal composite bone grafts combined with supplementary bioactive agents such as BMSCs or autograft have been shown to act synergistically to significantly improve fusion rates and bone quality compared to grafts composed of metals, autograft, or ceramics alone. Without adjunctive agents, strontium-containing bone grafts showed promise as independent osteoconductive materials, while magnesium-containing bone grafts had promising arthrodesis rates when combined with novel osteoinductive agents. The evidence for metal-related local or systemic toxicity or adverse events is limited in the literature describing metals incorporated in spinal fusion models. Future preclinical studies may serve to optimize specific metals or bioactive factors based on established osteoinductive mechanisms of action to maximize the arthrodesis rate achieved by these materials.

## Figures and Tables

**Table 1:** Designs and Investigated Outcomes of Included Studies.

Ref	Animal Model	Procedure	Levels Fused	Graft Composition	Graft Size	Sample Size (n)	Latest Follow-Up (Weeks)	Animals Fused at Latest Follow-Up (%)	Average (± SD) BV%	AE (n)	Histology of Fusion Masses
[24]	Goat	ACDF	C4–C5	(1) Porous Ta	10 mm × 10 mm	4	12	0	2.5 ± 4.6	1	Fibrous tissue only
				(2) Porous Ta + rhBMP-2	10 mm × 10 mm	4	12	25	11.0 ± 11.0	0	Bone deposition at implant site with metallic debris
[25]	Rabbit	PLF	L5–L6	(1) AG	2.0 g AG	15	8	60	NR	2	Trabecular bone deposition at implant site with fibrous interspace
				(2) Ti + AG	2.0 g AG + 200 mg Ti	15	8	60	NR	2	Bone deposition at implant site with dense fibrous tissue, inflammation, metallic debris, and apoptosis of tissue
[22,23]	Sheep	PLF + Discectomy	L3–L4 (n = 16) L4–L5 (n = 16)	(1) TiAlV Cage + AG	11 mm × 20 mm	16	52	25	37.6	0	Trabecular bone deposition with marked angiogenesis
				(2) Porous Ti-Ni	11 mm × 20 mm	16	52	81.25	25.4 (*p* = 0.052)	1	Trabecular bone deposition with dense fibrous tissue
[26]	Dog	ALIF	L5–L6	(1) Porous Ti	8 mm × 24 mm	5	12	60	40.8 ± 23.6	0	Incomplete bone deposition at implant site with dense fibrous tissue and marked angiogenesis
				(2) Porous Ti + NaOH-HCl	8 mm × 24 mm	5	12	100	81.9 ± 7.7 (*p* = 0.01)	0	Lamellar bone deposition at implant site with marked angiogenesis
[27]	Sheep	PLF	L2–L3 L5–L6	(1) AG	5 cm^3^ AG	16	24	62.5	NR	0	Bone deposition at implant site with sparse fibrous tissue
				(2) AG + Mg	5 cm^3^ AG + 1 cm^3^ Mg	16	24	81.25 (*p* = 0.119)	NR	0	Cortical and trabecular bone deposition with large marrow spaces
[28]	Sheep	PLF	T12–T13 L2–L3	(1) AG	5 cm^3^	18	24	“Partial”	55.9 ± 4.7	0	Trabecular bone deposition at implant site
				(2) MgHA	5 cm^3^	9	24	“Complete”	53.1 ± 5.6	0	Trabecular bone deposition at implant site
				(3) MgHA + DBM	5 cm^3^	9	24	“Partial”	44.3 ± 3.3 (*p* < 0.05 vs. AG)	0	Trabecular bone deposition at implant site
[29]	Rat	PLF	L4–L6	(1) Sr-TCP	NR	5	8	NR	25.6 (9.2) *	0	Trabecular bone deposition at implant site
				(2) Sr-TCP + BMSCs	NR	5	8	NR	24.0 (6.9) *	0	Trabecular bone deposition at implant site
				(3) Sr-TCP + ADSCs	NR	5	8	NR	26.0 (3.2) *	0	Poor bone deposition with dense fibrous tissue
[30]	Rat	PLF	L4–L5	(1) HA	NR	5	8	0	38.3	0	Bone deposition at implant site
				(2) HA + 7 mM ALN	NR	5	8	100	36.1	0	Bone deposition at implant site
				(3) HA + 28 mM ALN	NR	5	8	100	34.3	0	Trabecular bone deposition at implant site
				(4) SrHA 5%	NR	5	8	100	37.8	0	Trabecular bone deposition at implant site
				(5) SrHA 10%	NR	5	8	100	37.8	0	Trabecular bone deposition at implant site with marked endochondral ossification
[31]	Rabbit	PLF	L5–L6	(1) MgHA + Collagen	4 cm × 1 cm	12	6	NR	NR	0	Bone deposition at implant site with incomplete remodeling
[32]	Rabbit	PLF	L5–L6	(1) MgHA + Collagen	4 cm × 1 cm	8	6	12.5	NR	5	Trabecular bone deposition at implant site
				(2) MgHA + Collagen + PRP	4 cm × 1 cm	8	6	75	NR	0	Dense trabecular bone deposition at implant site
[33]	Rabbit	PLF	L5–L6	(1) AG	1.25 g	24	12	100	33.8 ± 1.6	0	Trabecular bone deposition at implant site with marked angiogenesis
				(2) Si-CaP	1.25 g	24	12	37.5	16.7 ± 0.8	0	Bone deposition at implant site with dense fibrous tissue and woven bone
				(3) Si-CaP + AG	0.625 g Si-CaP + 0.625 g AG	24	12	62.5	29.5 ± 1.4 (*p* < 0.05 vs. Si-CaP and Si-CaP + AG)	0	Trabecular bone deposition at implant site with marked angiogenesis
				(4) Si-CaP + AG + BMSCs	0.625 g Si-CaP + 0.625 g AG	24	12	100	32.5 ± 1.5	4	Trabecular bone deposition at implant site with marked angiogenesis; trace remnant Si-CaP
[34]	Rabbit	PLF	L4–L5	(1) AG	2 cc	18	26	87.5	68	0	Bone deposition at implant site with marked chondroblast tissue
				(2) Si-CaP + Hydrogel + AG	1 cm^3^ Si-CaP/Hydrogel + 1 cm^3^ AG	23	26	100	67	0	Trabecular bone deposition at implant site with marked endochondral ossification
				(3) Biphasic CaP + Collagen + AG	1 cm^3^ Biphasic CaP/Collagen + 1 cm^3^ AG	23	26	0 (*p* < 0.05 vs. AG and Si-CaP)	62	0	Bone deposition at implant site with dense fibrous tissue and autograft remnant
[35]	Rat	PLF	L4–L5	(1) AG	12 mm × 4 mm	20	8	100	49 ± 0.6	0	Bone deposition at implant site
				(2) AG + PLGA	12 mm × 4 mm	20	8	100	50.7 ± 0.5	0	Bone deposition at implant site
				(3) AG + PLGA + Mg	12 mm × 4 mm	25	8	100	51.1 ± 0.8	0	Bone deposition at implant site
				(4) AG + PLGA + Mg + DBM	12 mm × 4 mm	25	8	100	52 ± 0.6	0	Bone deposition at implant site
				(5) AG + PLGA + Mg + DBM + PDRN	12 mm × 4 mm	25	8	100	54.7 ± 1.2	0	Bone deposition at implant site with marked bone mineralization, calcium, and angiogenesis
[36]	Sheep	ACDF	C2–C3 C4–C6	(1) Valeo ceramic cage + AG	12 mm × 16 mm	8	6	100	NR	0	Implant-related histopathologic changes
				(2) Sr-Hardystonite- Gahnite	14 mm × 17 mm	8	6	100	NR	0	Implant-related histopathologic changes

SD = standard deviation, BV% = bone volume percentage, AE = adverse event, PLF = posterior lumbar fusion, HA = hydroxyapatite, AG = autograft, NR = not reported, ACDF = anterior cervical discectomy and fusion, Ta = tantalum, rhBMP-2 = recombinant human bone morphogenetic protein-2, TiAlV = titanium–aluminum–vanadium, ALIF = anterior lumbar interbody fusion, DBM = demineralized bone matrix, TCP = tricalcium phosphate, BMSC = bone marrow stem cell, ADSC = adipose stem cell, ALN = alendronate, PRP = platelet-rich plasma, CaP = calcium phosphate, PLGA = poly (lactic-co-glycolic acid), PDRN = polydeoxyribonucleotide. * denotes data reported as median values with interquartile ranges in parentheses.

**Table 2:** Systematic review center for laboratory animal experimentation (SYRCLE) risk of bias assessment.

Ref	Selection Bias			Performance Bias		Detection Bias		Attrition Bias	Reporting Bias	Was the Study Apparently Free of Other Problems that Could Result in High Risk of Bias
	Were Animals Randomly Assigned to Groups?	Were Groups Similar at Baseline?	Was Group Allocation Blinded?	Were the Animals Randomly Housed During the Experiment?	Were the Caregivers and/or Investigators Blinded from Knowledge of Which Intervention Each Animal Received During the Experiment?	Were Animals Selected at Random for Outcome Assessment?	Was the Outcome Assessor Blinded?	Were Incomplete Outcome Data Adequately Addressed?	Are Reports of the Study Free of Selective Outcome Reporting?	
[24]	Unclear	Yes	No	Yes	No	Yes	No	Yes	Yes	Yes
[25]	Yes	Yes	No	Yes	No	Yes	Yes	Yes	Yes	Yes
[22,23]	Unclear	Yes	No	Yes	No	Yes	No	Yes	Yes	Yes
[26]	Yes	Yes	No	Yes	No	Yes	No	Yes	Yes	Yes
[27]	Unclear	Yes	No	Unclear	No	Yes	Yes	Yes	Yes	Yes
[28]	Unclear	Yes	No	Yes	No	Unclear	No	Yes	No	Yes
[29]	Yes	Yes	No	Yes	No	Yes	Yes	Yes	Yes	Yes
[30]	Unclear	Yes	No	Yes	No	Yes	No	Yes	Yes	Yes
[31]	Yes	Yes	No	Yes	No	Yes	No	Yes	Yes	Yes
[32]	Unclear	Yes	No	Yes	No	Yes	Yes	Yes	Yes	Yes
[33]	Yes	Yes	No	Yes	No	Unclear	Yes	Yes	No	Yes
[34]	Unclear	Yes	No	Yes	No	Unclear	Yes	Yes	No	Yes
[35]	Unclear	Yes	No	Yes	No	Yes	No	Yes	Yes	Yes
[36]	Unclear	Yes	No	Yes	No	Unclear	No	Yes	No	Yes

## Data Availability

All data in this study is publicly available and may be requested from the authors.

## Abbreviations

The following abbreviations are used in this manuscript:

rhBMP-2	Recombinant human bone morphogenetic protein-2
TCP	Tricalcium-phosphate
HA	Hydroxyapatite
RANKL	Receptor activator of nuclear factor kappa ligand
PRISMA	Preferred reporting items for systematic reviews and meta-analyses
TP	Transverse process
BV%	Bone volume percentage
SYRCLE	Systematic review center for laboratory animal experimentation
ACDF	Anterior cervical discectomy and fusion
PLGA	Poly(lactic-co-glycolic acid)
DBM	Demineralized bone matrix
BMSC	Bone marrow stem cell
ADSC	Adipose stem cell
PRP	Platelet-rich plasma
PDRN	Polydeoxyribonucleotide
Ti-Al-V	Titanium–aluminum–vanadium
CaSR	Calcium-sensing receptor
PCL	Polycaprolactone
VEGF	Vascular endothelial growth factor

## Appendix A. Information Sources and Search Strategy

PubMed/MEDLINE was first manually queried without non-English language restriction from inception until 17 June 2025, using search terms related to metal composite bone grafts in animal models of spinal fusion. Search terms used were as follows: (“bone graft*”[tiab] OR “bone scaffold”[tiab] OR “graft”[tiab]) AND (“Spine”[mh] OR “spine”[tiab] OR “spinal”[tiab] OR “Spinal Cord”[Mesh] OR “spinal cord”[tiab] OR “lumbar”[tiab] OR “cervical”[tiab] OR “thoracic”[tiab] OR “sacral”[tiab] OR “Spinal Cord Compression”[Mesh] OR “Osteoarthritis, Spine”[Mesh] OR “Spinal Stenosis”[Mesh] OR “spinal stenosis”[tiab] OR “spine surgery”[tiab] OR “spinal surgery”[tiab] OR “Spinal Fusion”[Mesh] OR “spine fusion”[tiab] OR “spondylodesis”[tiab] OR “Laminectomy”[Mesh] OR “laminectomy”[tiab] OR “hemilaminectomy”[tiab] OR “Laminoplasty”[Mesh] OR “laminoplasty”[tiab] OR “foraminotomy”[tiab] OR “spine decompression”[tiab] OR “spinal decompression”[tiab] OR “spine arthrodesis”[tiab] OR “spinal arthrodesis”[tiab] OR “interbody fusion”[tiab]) AND (“metal*”[tiab] OR “Metals, Heavy”[Mesh] OR “Metals”[Mesh] OR “steel”[tiab] OR “magnesium”[tiab] OR “titanium”[tiab] OR “nickel”[tiab] OR “copper”[tiab] OR “cobalt”[tiab] OR “chromium”[tiab] OR “strontium”[tiab] OR “zirconium”[tiab] OR “tantalum”[tiab] OR “gold”[tiab] OR “silver”[tiab] OR “iron”[tiab] OR “platinum”[tiab] OR “zinc”[tiab] OR “lithium”[tiab] OR “manganese”[tiab] OR “silicon”[tiab] OR “silicon”[tiab]).
